# Image Processing for Bioluminescence Resonance Energy Transfer Measurement—*BRET-Analyzer*

**DOI:** 10.3389/fncom.2017.00118

**Published:** 2018-01-09

**Authors:** Yan Chastagnier, Enora Moutin, Anne-Laure Hemonnot, Julie Perroy

**Affiliations:** ^1^Centre National de la Recherche Scientifique, UMR-5203, Institut de Génomique Fonctionnelle, Montpellier, France; ^2^Institut National de la Santé Et de la Recherche Médicale, U1191, Montpellier, France; ^3^Universités de Montpellier, UMR-5203, Montpellier, France

**Keywords:** open source software, automatic image analysis, ratiometric measurements, bioluminescence resonance energy transfer

## Abstract

A growing number of tools now allow live recordings of various signaling pathways and protein-protein interaction dynamics in time and space by ratiometric measurements, such as Bioluminescence Resonance Energy Transfer (BRET) Imaging. Accurate and reproducible analysis of ratiometric measurements has thus become mandatory to interpret quantitative imaging. In order to fulfill this necessity, we have developed an open source toolset for Fiji—*BRET-Analyzer*—allowing a systematic analysis, from image processing to ratio quantification. We share this open source solution and a step-by-step tutorial at https://github.com/ychastagnier/BRET-Analyzer. This toolset proposes (1) image background subtraction, (2) image alignment over time, (3) a composite thresholding method of the image used as the denominator of the ratio to refine the precise limits of the sample, (4) pixel by pixel division of the images and efficient distribution of the ratio intensity on a pseudocolor scale, and (5) quantification of the ratio mean intensity and standard variation among pixels in chosen areas. In addition to systematize the analysis process, we show that the *BRET-Analyzer* allows proper reconstitution and quantification of the ratiometric image in time and space, even from heterogeneous subcellular volumes. Indeed, analyzing twice the same images, we demonstrate that compared to standard analysis *BRET-Analyzer* precisely define the luminescent specimen limits, enlightening proficient strengths from small and big ensembles over time. For example, we followed and quantified, in live, scaffold proteins interaction dynamics in neuronal sub-cellular compartments including dendritic spines, for half an hour. In conclusion, *BRET-Analyzer* provides a complete, versatile and efficient toolset for automated reproducible and meaningful image ratio analysis.

## Introduction

**Figure 1:**
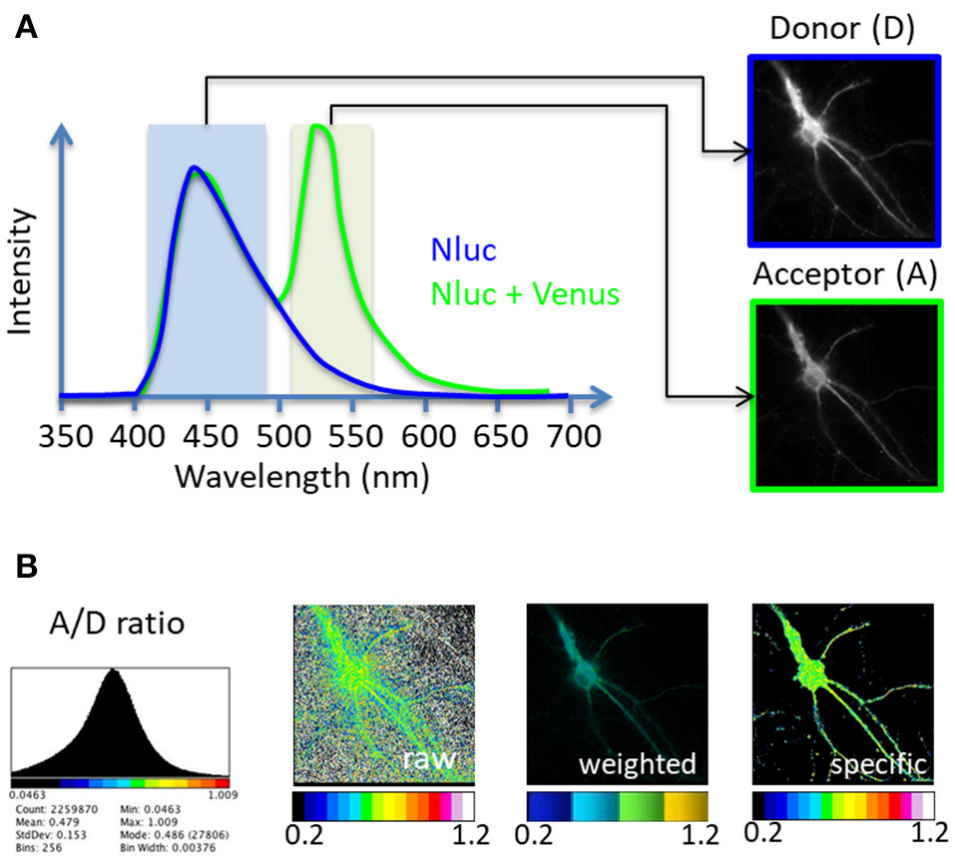
Principle of the BRET-Analyzer toolset. The BRET-Analyzer toolset provides automatic analyses of donor (D) and acceptor (A) images **(A)** to generate a quantitative analysis of the specific A/D ratio image **(B)**. **(A)** Scheme of two BRET donor compatible entities emission spectra: Nanoluciferase (Nluc) is the BRET donor and Venus the BRET acceptor. The light emitted by the donor alone displays an emission pic at 450 nm (blue curve). When Venus is co-expressed and close enough (<75Å) from Nluc, a non-radiative transfer of energy from Nluc excites Venus, which emits light at its characteristic wavelength (pic at 535 nm, green curve). Light collected with specific filters gives rise to donor and acceptor images. **(B)** Distribution of the A/D ratio pixel values and possible representations of the BRET images: A stack histogram (left panel) displays the distribution of the A/D ratio values obtained from pixel by pixel division of the A image over the D image, suggesting the minimal and maximal values to be set on the pseudo-color scale expressing the A/D ratio image. “Raw,” “weighted” and “specific” panels are three possible representations of the A/D ratio image. The “raw” image expresses the ratio as pseudo-colors, without any selection of pixels. Consequently, pixels with a near-zero intensity on the D image display a maximal A/D ratio, compromising the detection of pixels from the specimen. Alternatively, the “weighted” ratio also displays the A/D ratio intensity as pseudocolors, but the brightness of each pixel is proportional to the donor intensity. This representation shades the ratio background around the cell but also the specific signal coming from low protein expression level in sub-cellular area. The BRET-Analyzer toolset allows obtaining the “specific” ratio by an automatic selection of pixels from the specimen only (obtained by composite thresholding of the D image). The “specific” image displays the intensity of the A/D ratio expressed as pseudo-colors, regardless of protein expression levels.

A perpetual association and dissociation between proteins drives specific cellular signaling in time and space. Bioluminescence Resonance Energy Transfer (BRET) imaging is a sensitive technology to highlight the spatio-temporal dynamics of protein-protein interaction and understand their functions in intact living cells (Coulon et al., 2008; Perroy, 2010; Goyet et al., 2016; Faklaris et al., 2017). Briefly, the principle of the method stands on an energy transfer between a bioluminescent donor and a compatible fluorescent acceptor (Xu et al., 1999; Angers et al., 2000). By catalytic oxidation of its substrate, the bioluminescent donor emits light (donor image, D, Figure 1). Upon molecular proximity (<75Å), the BRET compatible acceptor is excited by a non-radiative transfer of energy and in turn emits light at its characteristic wavelength (acceptor image, A, Figure 1). Pixel by pixel division of the light emitted by the acceptor over the light emitted by the donor (A/D) gives rise to the BRET image, a ratiometric measurement expressed as pseudo-colors, allowing live quantification of the interaction between proteins tagged with BRET compatible entities, in subcellular domains (Goyet et al., 2016). The advantage of BRET over other RET methodologies precisely comes from the absence of light to initiate the energy transfer in BRET. Thereby, BRET circumvents many drawbacks linked to light excitation (such as auto-fluorescence of cells, direct excitation of the acceptor fluorophore by the donor exciting light, or photobleaching of fluorophores) giving rise to an excellent signal over noise ratio.

A ratiometric measurement provides the huge benefit to normalize the output signal to the intensity of its stimulus. Hence, any efficient activation of a system can virtually be reported, regardless of the stimulus intensity. This is particularly relevant to put emphasis on effective forces of small ensembles. However, this benefit is counterbalanced by the fact that a near-background stimulus will give rise to an aberrant ratiometric measure resulting from the fraction's near-zero denominator. Consequently, the specific signal is lost in a high-intensity background. Therefore, delimitation of the linear detection range of the recording system and proper determination of the stimulus intensity threshold is mandatory to perform ratiometric measurements. In the present work, we have developed a systematic image processing to obtain relevant ratiometric measurements. We applied this automatic processing on BRET images. This homemade toolset is a complete open source solution for Fiji [free software for scientific image analysis, (Schindelin et al., 2012)], available for the scientific community together with a step-by-step tutorial (https://github.com/ychastagnier/BRET-Analyzer).

The *BRET-Analyzer* toolset includes classical processing of the D and A images by cleaning the background, and pixel by pixel division of the A/D images. We worked mainly on two parameters. First we defined a donor threshold computed as a function of the donor signal intensity in a given area. This step allows defining the frontiers between the sample luminescence *per se* and the light spread on neighboring pixels. Second we selected and combined different threshold processes independently in each sub-area, depending on the local donor intensity. This is particularly relevant when the recorded cell displays heterogeneous subcellular volumes. For example in neurons, a cytosolic signal from the donor accumulated in the soma will be much brighter than the donor light recorded in thin neuritic processes. A combination of donor threshold processing allows proper reconstitution of the ratiometric image. It is fundamental to note that getting rid of low-level donor pixels permits the selection of pixels from the luminescent specimen only, but does not influence the ratiometric measurement *per se*. We thus provide here a toolset—*BRET-Analyzer*—that allows the elimination of non-specific signal, and performs quantitative analysis of long period recordings of protein-protein interactions, regardless of the protein expression level (Figure 1).

## Stepwise procedures and anticipated results

Raw images of the donor (D) and acceptor (A) BRET entities were obtained as previously described (Goyet et al., 2016), from hippocampal neurons (Figure 1). To summarize the process, the images will first have their background subtracted, then be aligned over time. A threshold will be computed to separate signal from background. A image is divided by D image pixel by pixel. Finally, measures are made on the ratiometric images obtained.

The analysis starts with the removal of non-specific signals (“*clean*” button, Supplementary Material 4.1), in D and A images, in 3 simple steps. First, in each image, applying a median filter of radius 1 (3^*^3 pixels) allows to remove outliers. Second, subtracting the median value of a region corresponding to the background removes the camera offset and global light background, assuming they are homogeneous. The homogeneous assumption is based on the fact that BRET doesn't use illumination, unlike fluorescence based imaging. Subtracting a background image is available as an option. This value is measured on each image of the donor and the acceptor, to account for potential fluctuations of the background's mean intensity from an image to another. Third, in case recording is a timelapse, aligning images might be needed. To do so, we make use of the plugin TurboReg (http://bigwww.epfl.ch/thevenaz/turboreg/ Thevenaz et al., 1998), a pyramid approach to subpixel registration based on intensity. This alignment can rescue xy drift that may happen during multi-positioning of the microscopy setup to record distant cells on the same sample. It is important that images are aligned through time to make sure the area in which the measures are made always corresponds to the same part of the sample.

Second step is to divide, pixel by pixel, the A image by the D image (“*divide*” button, Supplementary Material 4.3). Prior to this division, it is necessary to get rid of all pixels that have an intensity level too close to the noise level on the donor image, to keep only specific signal from the sample (Figure 1B, “*specific”*). Indeed, the computed A/D ratio tends to infinity for each pixel with a near-zero intensity on the D image. If these pixels are not removed, the ratiometric values can range from zero to hundreds and the boundaries of the cells cannot be distinguished (Figure 1B, “*raw”*). Alternatively, the brightness of the A/D ratio image can be weighted by the expression level of proteins (Figure 1B, “*weighted”*), which efficiently hides non-specific pixels, but also fades ratiometric values from small protein numbers. This weighting can be attractive for homogenous samples but should be avoided to perform ratio-metric measurements from heterologous volume specimens since it overshadows proficient strengths from small ensembles.

**Figure 2:**
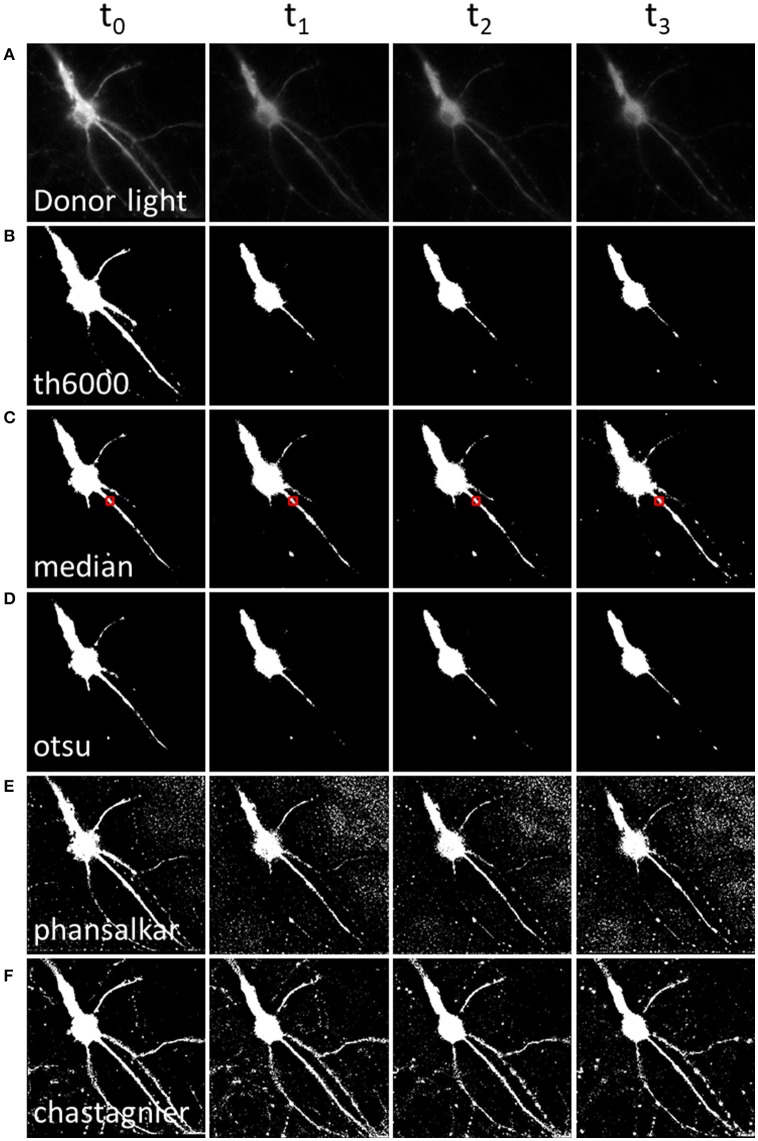
Methods to define a donor threshold in space and time. Applying a threshold to the donor image before division allows masking irrelevant pixels (background around the luminescent specimen) in the A/D ratio image. Various threshold methods **(B–F)** can be applied to the donor image **(A)**. Each column represents a different time point. **(A)** Intensity of the donor for each time point. **(B)** Mask using a constant threshold all over the image time series. **(C,D)** Mask using a global threshold computed for each time point (median threshold of the area outlined in red, **(C)** or Otsu thresholding method, **(D)**. **(E,F)** Mask using a local threshold computed for each time point, Phansalkar **(E)** or Chastagnier threshold **(F)**, described in Figure 3. Note that computing a local threshold for each image over the time series allows to precisely define the luminescent specimen limits, all along the experiment.

Hence, in order to remove pixels that are outside of luminescent specimen from the resulting A/D ratio image, we applied available threshold methods to the D image (Figure 2). The simplest approach, applying a static threshold level of a given value across all images (Figure 2B) presented two main problems. First, since the D signal decreases with time, part of the specific D signal falls below the threshold during image time series when the threshold was determined on the first image. Conversely, lowering the threshold resulted in keeping the noise on the first images of the image time series. We circumvented this problem by computing a specific threshold for each individual image of the image time series. We selected a region of interest (which should contain about half pixels to keep and half to remove). For each time point, we computed the median of that area as the threshold to be applied on the D image (Figure 2C). Alternatively, we used an automatic threshold built in Fiji. Across all the available automatic thresholding methods, Otsu [which searches for the threshold value that minimizes the intra-class variance (Otsu, 1979)] gave the best results at separating the high signal from the rest (Figure 2D).

However, a second problem was related to the fact that, for a given time point, the D image displayed strong intensity differences among neuronal subareas (Figure 2A). Therefore, applying a homogenous constant threshold on the D image did not allow suppressing pixels adjacent to the neuronal cell body (which, even so located out of the neuron, still displayed a relatively high light intensity due to the D light spread), while keeping the low but specific D light from neuritic processes (Figures 2B–D). To solve this spatial problem, one possibility was to crop different regions of interest (“crop” button, Supplementary Material 4.2) and compute a specific threshold for each crop. But the resulting BRET image of the neuron was segmented in several areas. Furthermore this process increased analysis time to quantify the BRET images (step below), as it required a repeated exploration on each crop. We thus favored a second analysis process to determine the threshold of the D image in space. Automatic local thresholds built in Fiji have the advantage to compute the threshold for each pixel based on the surrounding pixels. Hence values that are locally high are selected, allowing inhomogeneous signal across the image. For example Phansalkar threshold (Phansalskar et al., 2011), provided good results at removing noise at the edges of cells making clear delimitation, but kept a good part of the noise far from the cells (Figure 2E).

**Figure 3:**
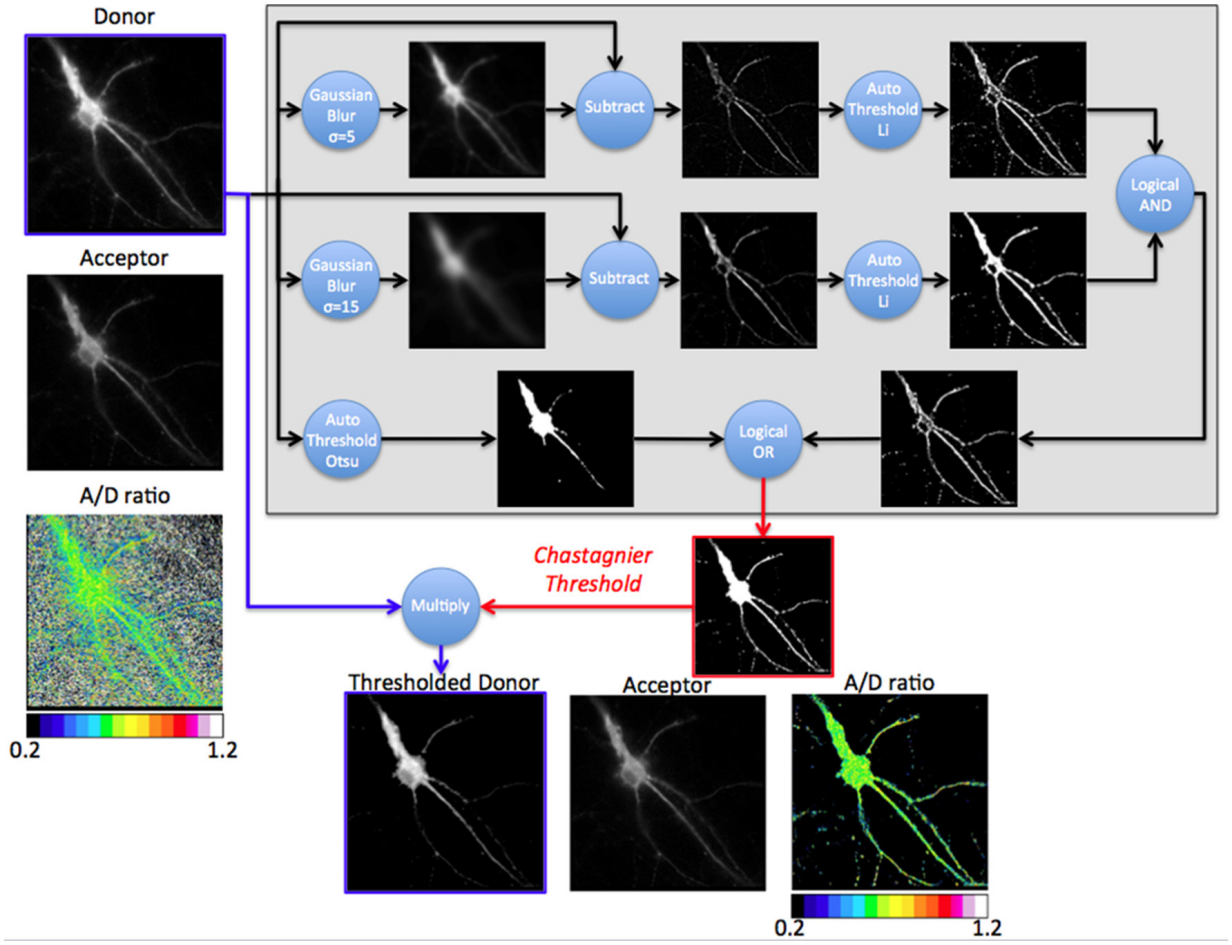
Principle of the Chastagnier threshold. The Chastagnier threshold computed on the donor image (Gray rectangle) creates the mask (red image) to be applied on the donor image (thresholded donor) prior to A/D images division. Left column shows the donor, acceptor and A/D ratio images without mask. The gray rectangle is a diagram representation of the process used for Chastagnier thresholding method. A composite process using Gaussian blur subtraction and Li's thresholding method identifies pixels with locally high intensity. In parallel, Otsu's thresholding method preserves pixels with globally high intensity. A logical OR combines the two processes to obtain the final mask. This mask (red image) with values of 0 for black pixels and 1 for white pixels multiplies the original D image to remove irrelevant pixels without affecting the donor light intensity of pixels from the fluorescent specimen (Thresholded donor). Bottom line shows the thresholded donor, the acceptor and resulting A/D ratio images.

We thus designed our own composite thresholding method (Chastagnier threshold, Figure 2F). The whole process consisted in drawing an accurate mask around the neuron to keep only pixels of the D image arising from the luminescent specimen. To create this mask (Figure 3, gray rectangle), we first subtracted a blurred version of the D image to itself in order to increase the contrast, and convert it to binary values using Li's automatic threshold method (Li and Tam, 1998). When the blur effect is decreased, noise areas start to show up, while when blur effect is increased, areas of interest show up thicker than they really are. To get rid of the defects of both low and high blur effect, we used both and combined them with a logical AND in order to keep only pixels that are in both images. As “holes” appeared in the lowest intensity area of two juxtaposed regions of high intensity areas, we combined it using a logical OR with the original image on which Otsu Threshold was applied (to keep only high signal). Logical OR is inclusive, so it keeps pixels that are in an image, in the other, or in both. We thus obtained a binary image with values 0 and 255. Dividing it by 255 gave 0 for pixels we want to remove and 1 for pixels to keep. This is the mask (Figure 3, Chastagnier Threshold). Multiplying it with the original D image removed irrelevant pixels (set them to 0) and did not affect the value for the rest of the D image (Figure 3, Thresholded donor). The Chastagnier's threshold thus unequivocally refined the limits of the luminescent sample in space and time. For user convenience, we nevertheless included the conventional alternative thresholding methods herein tested, as an option in the *BRET-Analyzer* toolset.

The A image was then divided by the thresholded D image and displayed with a 16 pseudo-colors look up table, ranging from cold to hot colors (Supplementary Material 4.3.5). The minimal and maximal A/D ratio values between which the colors will be distributed have to be selected. The stack histogram displayed between the 5th and 995th permilles (without taking into account pixels with value 0) can be used to help the user choose appropriate values (Figure 1B). Once the range is set, it should be kept the same for every image, so they can be visually compared, before doing the in depth analysis. The resulting BRET image is a quantitative measurement of the energy transfer intensity reporting either the efficiency of interaction between two proteins tagged with BRET compatible entities (intermolecular BRET) or conformational changes of a molecule tagged with D and A entities (intramolecular BRET). This analysis displays BRET intensity regardless of the level of protein expression. Hence, even small numbers of complexes are measured and visualized on the BRET image. This provides an important benefit compared to ratiometric analysis in which the signal is expressed as discontinuous pseudo-color scale representing two different parameters in one, namely A/D ratio and expression level (the brightness of the image being weighted by the protein expression level, e.g., by the D and/or A image intensity). This is potentially confusing and may lead to arbitrary judgments about the extent of BRET. Indeed, this kind of analysis, presented on Figure 1 as “*weighted”* A/D ratio allows to visually exclude the noise but the evidenced BRET signals come from proteins with high expression level only, neglecting the functional importance of high protein interactions of small ensembles. The weighted representation is nevertheless included as an option in the *BRET-Analyzer* (Supplementary Material 4.5 Param Tool, Display weighted images?).

**Figure 4:**
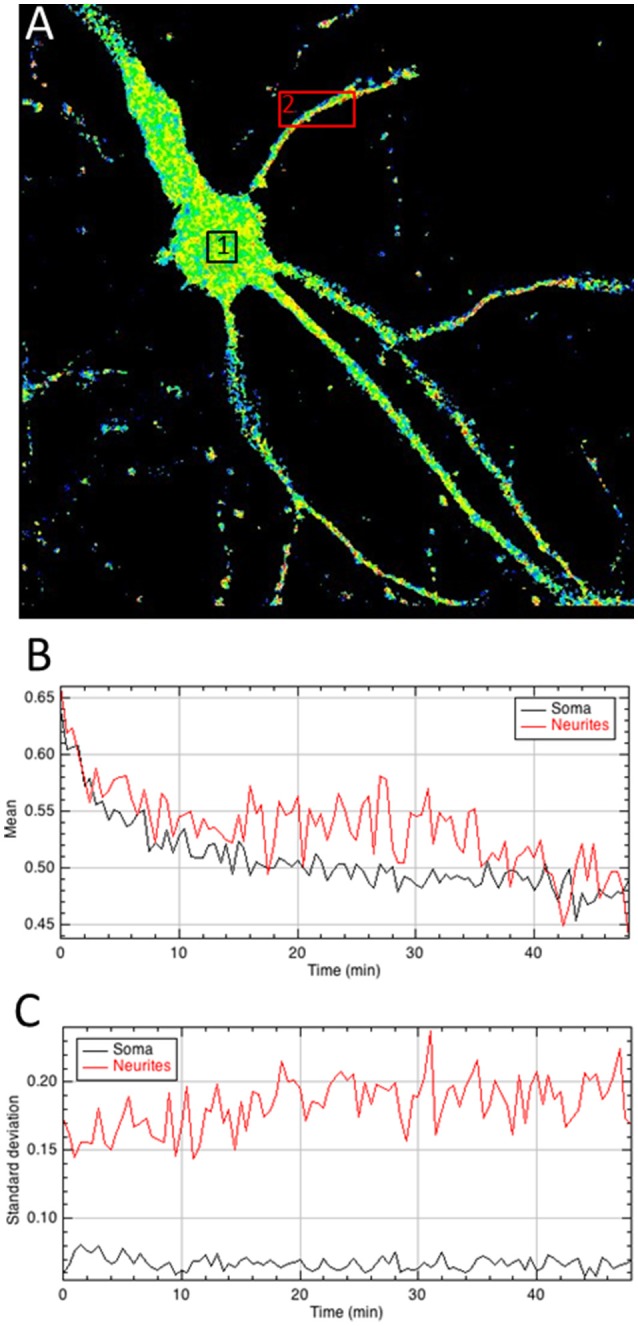
Quantitative analysis of the A/D ratio image**. (A)** Regions of interest are drawn on the ratiometric image. **(B,C)** The mean intensity of pixels within areas of interest quantifies BRET signals over time **(B)** whereas the standard deviation of A/D ratio between pixels **(C)** provides a measurement of the homogeneity of the BRET signal within areas and the evolution as a function of time.

Final step is to extract the BRET values out of the ratiometric image, in regions of interest, which can be chosen on a single image (Figure 4), or pooled from multiple images (Supplementary Material 4.4). All tools can be used to define the area of interest (rectangle, polygon, freehand…), at user convenience. It is not necessary to precisely draw the limits of the samples with the tool, because all pixels with a null value are being automatically ignored by the measures and excluded from the analysis of the BRET quantification in the area. The mean BRET intensity provides a quantification of the number of protein complexes in a given area and the standard deviation of the BRET intensity between pixels reports homogeneity or clusterization of the BRET signal.

**Figure 5:**
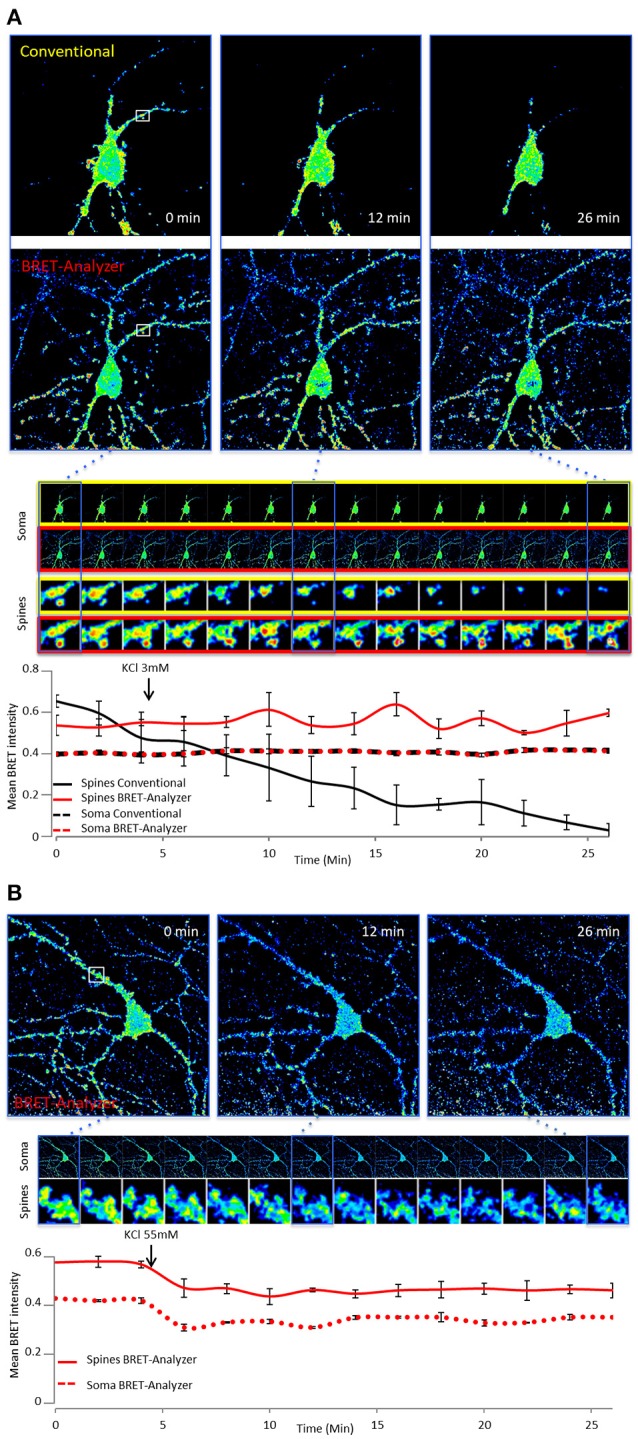
Ratiometric measurements in heterogeneous subcellular volumes using BRET-Analyzer. Hippocampal neurons expressing mGlu5 and Homer proteins fused to BRET compatible entities were recorded as previously described (Moutin et al., 2012; Guo et al., 2015) for 30 min. Images were analyzed using conventional (A) and /or BRET-Analyzer protocols **(A,B)**. **(A)** Images obtained in control condition (KCl 3 mM) were analyzed twice, using the conventional BRET analysis (Yellow-framed pictures) or the BRET-Analyzer (Red-framed pictures) protocol. We quantified the mean BRET intensity in soma and dendritic spines over time. Note that BRET signals measured by BRET-Analyzer are stable in all cellular compartments for half an hour. By opposition, the conventional analysis failed to quantify the BRET in spines over time. **(B)** BRET-Analyzer enables live, BRET image time series of mGlu5-Homer interaction in neurons. We quantified the mean BRET intensity in single areas drawn on soma and dendritic spines over time, before and during neuronal depolarization (KCl 55 mM). KCl 55 mM decreases BRET signals in soma and spines. BRET-Analyzer enables precise measurements of ratiometric signals over time. Each point of the curves is the mean ± SEM of BRET intensity recorded in 3 areas.

In addition to the automation of analysis process, the main strength of *BRET-Analyzer* resides in the potency to quantify luminescent ratio regardless of protein expression levels. This is particularly relevant when working on heterogeneous subcellular volumes such as neurons. Indeed, a neuron contains a large range of volumes from the neuronal cell body (around 500 μm^3^) to small dendritic spines (spine head volumes ranging from 0.01 μm^3^ to 0.8 μm^3^). The first challenge, as discussed before is thus to find an adequate composite threshold allowing to perform ratiometric measurements in all volumes at the same time. Moreover for the smallest compartments, measuring protein-protein interactions from few proteins is difficult and live dynamics over time are even more challenging. To illustrate the benefits from the herein developed analysis toolset, we analyzed twice the same images either with the conventional analysis (including a static threshold level of D images, described in Figure 2B) or with *BRET-Analyzer* (using Chastagnier's threshold, Figure 2F). We thus measured in neurons the interaction between a glutamate receptor, mGlu5, and its cognate scaffold, Homer, together involved in neuronal synaptic transmission (Moutin et al., 2012; Guo et al., 2015). As shown in Figure 5A, the interaction between mGlu5 and Homer was accurately recorded over time in the soma and the quantification indiscernible whatever was the analysis protocol. However, the advantage of using *BRET-Analyzer* became obvious for small processes, which from the beginning of the experiment were barely identifiable using the classical analysis protocol and totally lost few minutes after. *BRET-Analyzer* enables long time recording of BRET signals in small subcellular volumes, allowing for the first time live and stable measurements of mGlu5-Homer interaction in individual spines for half an hour. By opposition to previous studies (Moutin et al., 2012; Guo et al., 2015), instead of assessing synaptic activity-induced changes in mGlu5-Homer interactions in spines from two distinct cell populations (stimulated or not), we could here follow BRET changes using *BRET-Analyzer* in the same spines before and during KCl-induced neuronal depolarization. Thus, neuronal depolarization disrupted mGlu5-Homer interaction (Figure 5B). We recorded a 15 and 16% drop of BRET signal in soma and spines, respectively. This BRET decrease was measured as soon as 2 min following KCl 55 mM perfusion and stable for 20 min. Hence, *BRET-Analyzer* enables image time series of ratiometric measurements even in small cellular compartments.

## Materials

### Source code

The data were analyzed using a toolset made for Fiji (https://fiji.sc) (Schindelin et al., 2012). We here provide a step-by-step tutorial as Supplementary Material. The toolset and documentation details of how to use it are also publicly available at (https://github.com/ychastagnier/BRET-Analyzer), and will be regularly updated. Moreover, the tools can be freely downloaded, modified and improved to fit future research needs.

TurboReg, the plugin used to align stacks of images can be downloaded at (http://bigwww.epfl.ch/thevenaz/turboreg/) (Thevenaz et al., 1998).

### Example luminescent images

The donor and acceptor fluorescent images used here to exemplify the use of *BRET-Analyzer* corresponds to an hippocampal neuron from primary cell culture transfected with an intramolecular BRET-based sensor for ERK activity [YEN (Goyet et al., 2016)], except in Figure 5, where mGlu5-NanoLuc and Venus-Homer intermolecular BRET was recorded. Raw images are available at https://github.com/ychastagnier/BRET-Analyzer.

## Author contributions

YC designed the Fiji BRET-Analyzer plugin, computed all steps of the automated image analysis protocol and wrote the manuscript and step by step tutorial. EM and A-LH performed BRET imaging experiments. JP conceived, designed, led the project, supervised the analysis strategy and wrote the manuscript.

### Conflict of interest statement

The authors declare that the research was conducted in the absence of any commercial or financial relationships that could be construed as a potential conflict of interest.

## References

[B1] Angers S. Salahpour A. Joly E. Hilairet S. Chelsky D. Dennis M.. (2000). Detection of beta 2-adrenergic receptor dimerization in living cells using bioluminescence resonance energy transfer (BRET). Proc. Natl. Acad. Sci. U.S.A. 97, 3684–3689. 10.1073/pnas.060590697 10725388 PMC16300

[B2] Coulon V. Audet M. Homburger V. Bockaert J. Fagni L. Bouvier M.. (2008). Subcellular imaging of dynamic protein interactions by bioluminescence resonance energy transfer. Biophys. J. 94, 1001–1009. 10.1529/biophysj.107.117275 17921204 PMC2186264

[B3] Faklaris O. Heuninck J. Falco A. Goyet E. Zwier J. M. Pin J.-P. (2017). Fluorescent-based strategies to investigate G protein-coupled receptors: evolution of the techniques to a better understanding, in Topics Medicinal Chemistry (Berlin; Heidelberg: Springer International Publishing).

[B4] Goyet E. Bouquier N. Ollendorff V. Perroy J. (2016). Fast and high resolution single-cell BRET imaging. Sci. Rep. 6:28231. 10.1038/srep28231 27302735 PMC4908377

[B5] Guo W. Ceolin L. Collins K. A. Perroy J. Huber K. M. (2015). Elevated CaMKIIalpha and hyperphosphorylation of homer mediate circuit dysfunction in a Fragile X syndrome mouse model. Cell Rep. 13, 2297–2311. 10.1016/j.celrep.2015.11.013 26670047 PMC4685008

[B6] Li C. H. Tam P. K. S. (1998). An iterative algorithm for minimum cross entropy thresholding. Pattern Recogn. Lett. 18, 771–776. 10.1016/S0167-8655(97)00051-2

[B7] Moutin E. Raynaud F. Roger J. Pellegrino E. Homburger V. Bertaso F.. (2012). Dynamic remodeling of scaffold interactions in dendritic spines controls synaptic excitability. J. Cell Biol. 198, 251–263. 10.1083/jcb.201110101 22801779 PMC3410417

[B8] Otsu N. (1979). A threshold selection method from gray-level histograms. IEEE Trans. Syst. Man Cyber. 9, 62–66. 10.1109/TSMC.1979.4310076

[B9] Perroy J. (2010). Subcellular dynamic imaging of protein-protein interactions in live cells by bioluminescence resonance energy transfer. Methods Mol. Biol. 591, 325–333. 10.1007/978-1-60761-404-3_19 19957139

[B10] Phansalskar N. More S. Sabale A. Joshi M. (2011). Adaptive local thresholding for detection of nuclei in diversity stained cytology images. in International Conference on Communication and Signal Processing, 218–220.

[B11] Schindelin J. Arganda-Carreras I. Frise E. Kaynig V. Longair M. Pietzsch T.. (2012). Fiji: an open-source platform for biological-image analysis. Nat. Methods 9, 676–682. 10.1038/nmeth.2019 22743772 PMC3855844

[B12] Thevenaz P. Ruttimann U. E. Unser M. (1998). A pyramid approach to subpixel registration based on intensity. IEEE Trans. Image Process. 7, 27–41. 10.1109/83.650848 18267377

[B13] Xu Y. Piston D. W. Johnson C. H. (1999). A bioluminescence resonance energy transfer (BRET) system: application to interacting circadian clock proteins. Proc. Natl. Acad. Sci. U.S.A. 96, 151–156. 10.1073/pnas.96.1.151 9874787 PMC15108

