# Supplementary material for: Image Processing for Bioluminescence Resonance Energy Transfer Measurement—BRET-Analyzer
Source: Front Comput Neurosci. 2018 Jan 9;11:118. doi: 10.3389/fncom.2017.00118 (PMC5767221; doi:10.3389/fncom.2017.00118)
Supplement: Supplementary file 1 [file DataSheet1.PDF]

BRET Analyzer toolset for Fiji

Yan Chastagnier

December 18, 2017

Contents

1 Setup **2**

2 Tools description **2**

3 Image naming convention **3**

4 Tools use **4**

4.1 Clean Tool 4

4.2 Crop Tool 5

4.3 Divide Tool 6

4.4 Analyse Tool 9

4.5 Param Tool 13

4.6 Min Max Tool 14

5 Output files structure **14**

1 Setup

Put .ijm file into the folder Fiji.app/macros/toolsets/ (on MacOS X, right click Fiji.app > Show content folder).

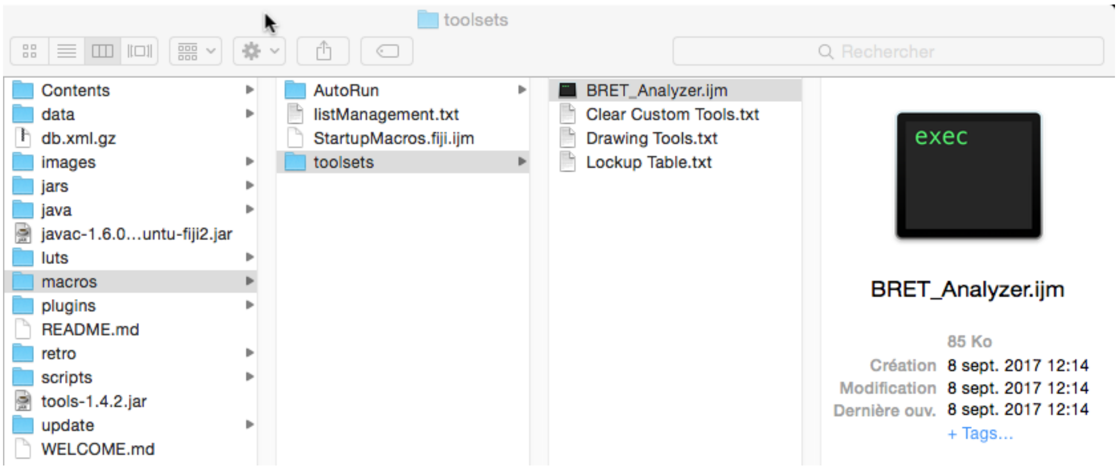

Figure 1 – Location to put .ijm file.

Click on » symbol at the extrem right of the toolbar then select the toolset to display the tools.

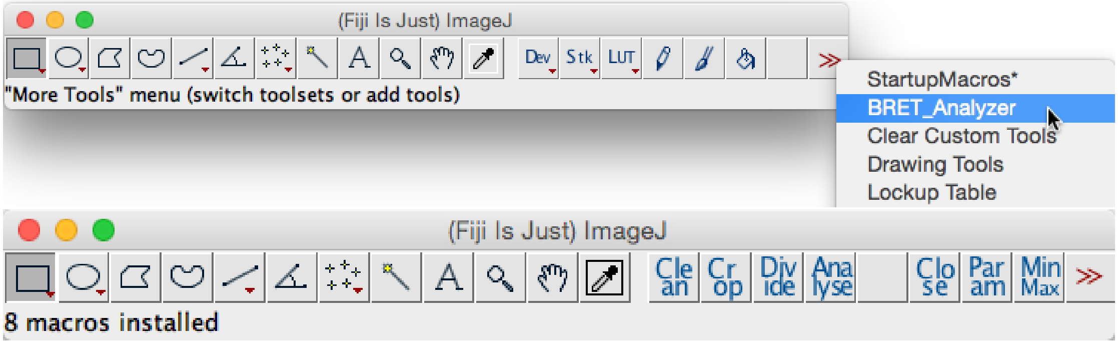

Figure 2 – Display the tools.

2 Tools description

2.1 Clean tool

Apply a median filter of radius 1.

Remove the background signal by subtracting the median value of a region that contains only noise. Optionally subtract a background image to remove inhomogeneous background.

Align the stacks to correct the small xy drift due to the multipositioning of the microscope by computing the translations coordinates for a stack of images and applying it to both (optional).

2.2 Crop tool

Extract sub regions to analyse them separately with different thresholds (should be used only on last resort if no thresholding method works well).

2.3 Divide tool

Set to 0 all points below a computed dynamic threshold on the clean donor stack of images. The threshold can be obtained using different methods. Automatic Threshold methods which compute the threshold level based on the whole image (or stack). Automatic Local Threshold methods compute threshold for each pixel based on the surrounding pixels in a given radius. Manual methods: selecting an area and calculating its median or mean * coefficient. In addition to one of the previous methods, a *Minimum threshold* can be set.

Divide pixel by pixel the acceptor by the donor, to obtain ratiometric images.

Set the image to 16-colors and adjust the range of values to visualize it in pseudo-colors.

2.4 Analyse tool

Select regions of interest and measure the mean and standard deviation on each slice.

Plot data for each regions.

2.5 Close tool

Close ROI Manager, Results window, Log window and all images.

2.6 Param tool

Change behavior of other tools and set default parameters.

2.7 Min Max tool

Allows to change the visualisation range of all "Ratio" images in a folder and its subfolders, in order to have an homogeneous distribution across images.

3 Image naming convention

It is suggested to name raw image files xxxDonorName.tif and xxxAcceptorName.tif, where xxx can be anything as long as it's the same for donor and acceptor images. DonorName and AcceptorName can be anything as well, but they have to be entered into the fields "Donor" and "Acceptor", in the upper lines of the parameter box. This way, when choosing the donor, acceptor will be automatically found and both will be opened (this also works if DonorName and AcceptorName are in the middle of the image names). This is a necessary step to do batch processing.

It is possible to use any name, but both donor and acceptor will have to be selected, and the subfolder will be the donor name without .tif extension, if "DonorName" is not found in the file name. The DonorName and AcceptorName (which are in the fields "Donor" and "Acceptor" in the parameters) will be used in the names of the clean images.

4 Tools use

4.1 Clean Tool

1. Select raw donor image (and raw acceptor image if necessary).

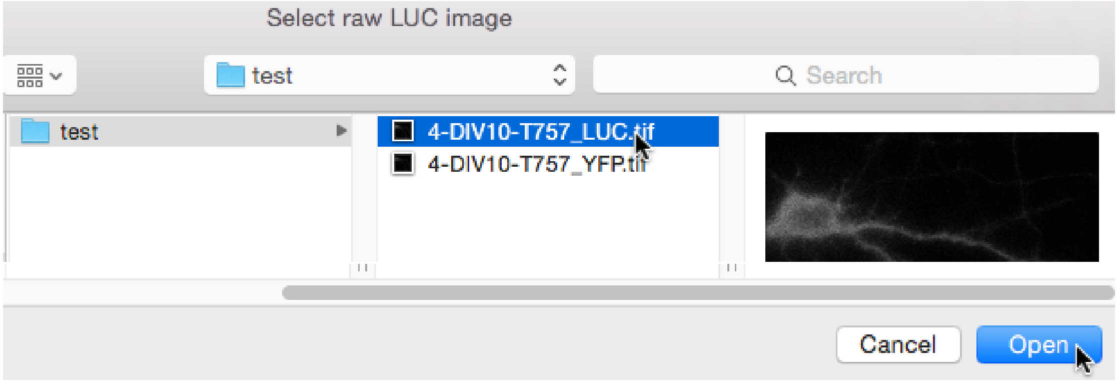

Figure 3 – Window to select raw donor image.

2. If *Subtract background image?* is ticked in the parameters, select donor then acceptor background images.
3. Select background area then click "OK".

   A 32*32 pixels area minimizing mean value over the stack is automatically selected. If this value seems always good, consider ticking *Batch clean?* in the parameters to speed up the process.

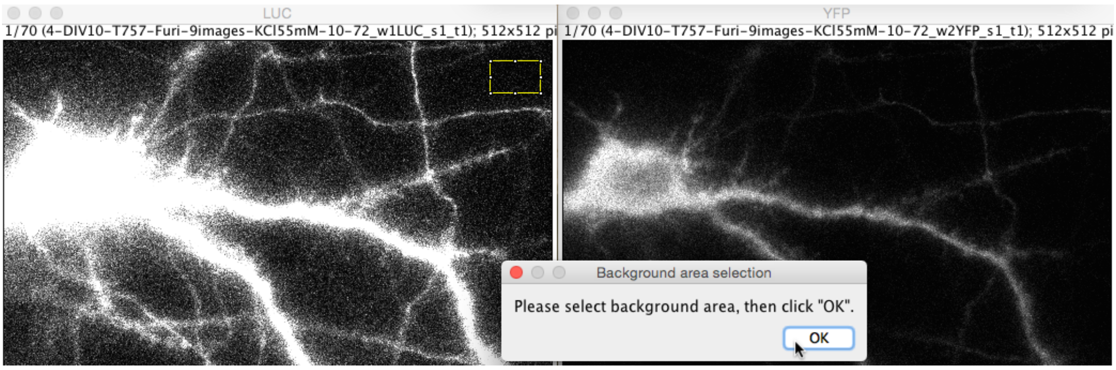

Figure 4 – Background area selection.

4. Choose if you want to align images in the stack or not. If you do, check the result at the end of the clean step. It might fail if there is a low contrast and a drift may appear on long stacks.

   Note: TurboReg, the plugin used to register the movement seems not to be part of the latest update of Fiji. If it is missing, you will have to download from http://bigwww.epfl.ch/thevenaz/turboreg/ the package for your distribution. Unpack it, place the file TurboReg_.jar into Fiji.app/plugins/ and restart Fiji.

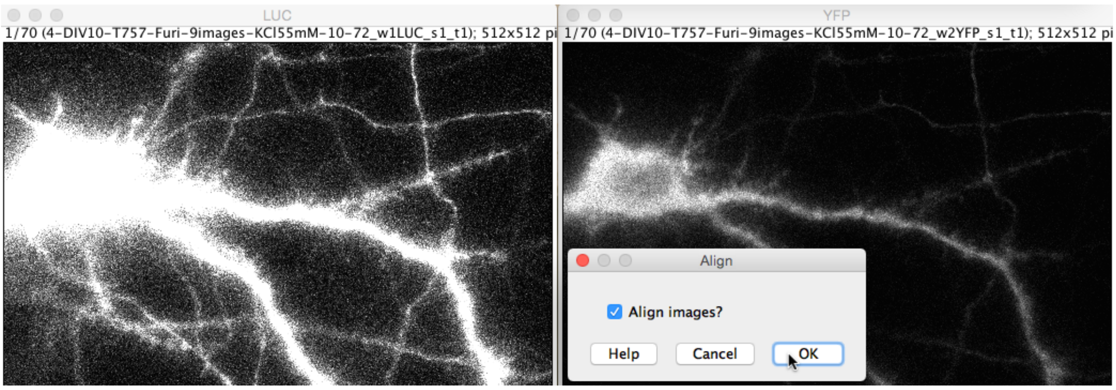

Figure 5 – Choose to align images or not.

5. Images are processed then saved in a subfolder.

Note: If *Batch clean?* is ticked in the parameters, replace all steps by "Select the directory containing raw images". All the pairs of images following naming convention found in the folder will be processed with default parameters.

4.2 Crop Tool

1. If images are not already open, select donor image (and acceptor image if necessary).
2. Select a rectangular area to crop, then click "OK".

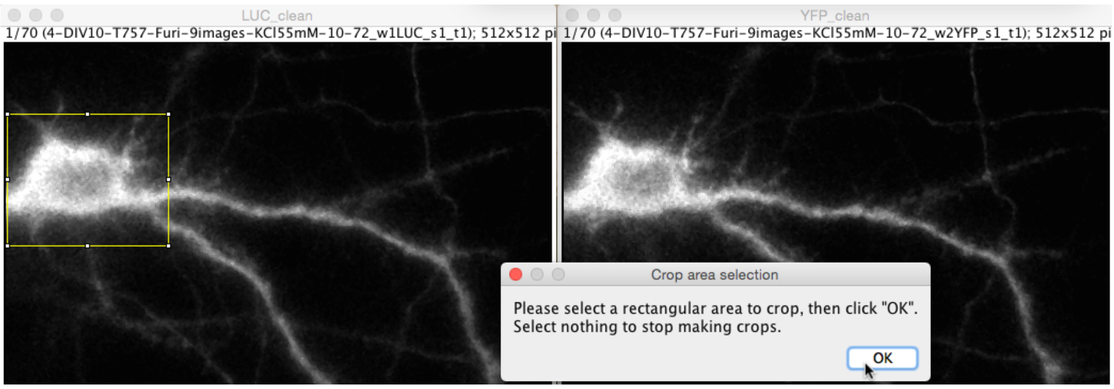

Figure 6 – Crop area selection.

3. Enter a label for the crop then click "OK" (this step is skiped if *Give a label to CROPs?* is unticked in parameters).

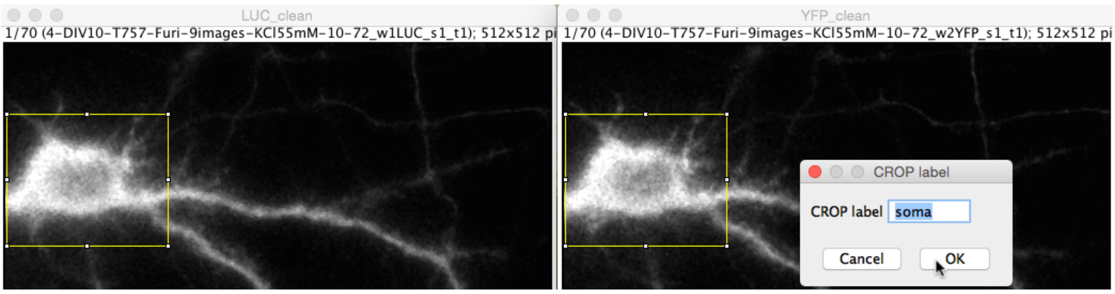

Figure 7 – Give a label to crop.

4. Select nothing then click "OK" to stop making crops.
5. Images are saved then closed.

Note: If *Batch crop?* is ticked in the parameters, replace step 1 by "Select the directory containing raw images", then apply other steps for each pair of images.

4.3 Divide Tool

1. If images are not already open, select donor image (and acceptor image if necessary).
2. If threshold method chosen in "parameters" is "Median" or "Mean * coefficient", select area then click "OK".
3. The threshold is computed and applied for each slice and displayed in a new window, named "<DonorName>_test". If the threshold method is AutoTh-Otsu or AutoTh-Chastagnier, another window, named "<DonorName>_Mask" displays the mask used for the threshold. Note that the *Minimum threshold* (see Figure 9) is applied after the mask. It means that pixels with value below the *Minimum threshold* that are on the mask won't be in the final image. Check if the result is good then click "OK".

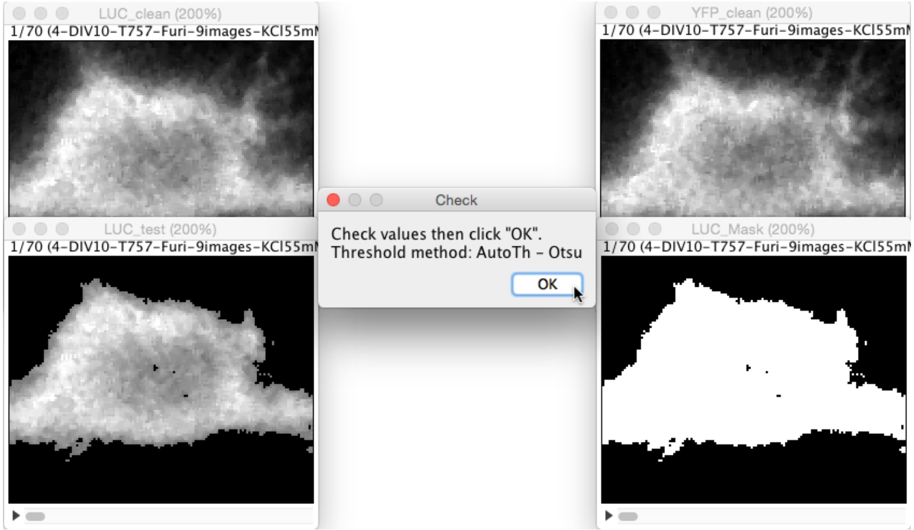

Figure 8 – Check threshold. Bottom left, donor image on which the threshold has been applied. Bottom right, the mask generated by AutoTh methods only.

4. Adjust threshold parameters if needed. Keep *Update Threshold Settings?* ticked to start thresholding process again with new parameters. Untick it to keep settings and continue.

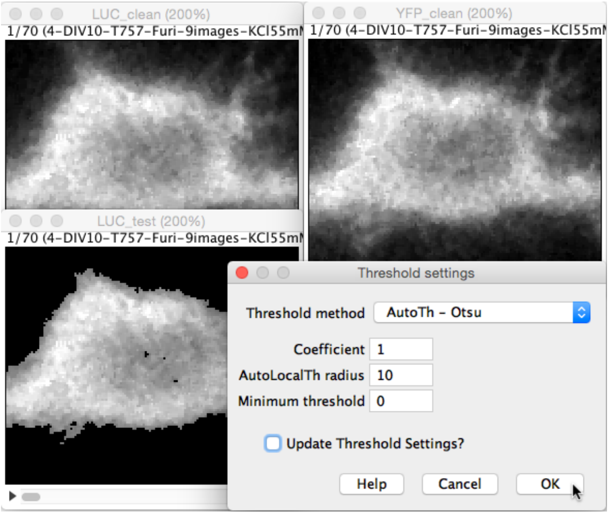

Figure 9 – Threshold settings.

5. Ratiometric image is obtained dividing acceptor by donor. It is displayed in 16 colors. Check the color range (click Live on slice histogram to see distribution along the stack, or use the stack histogram), then click "OK".

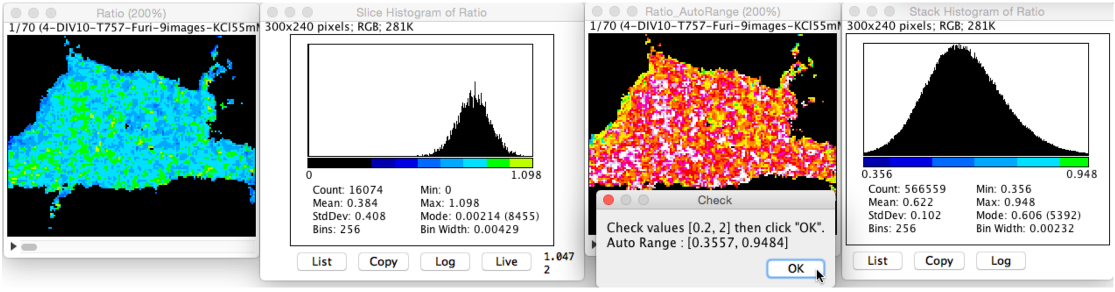

Figure 10 – Check color range.

6. Adjust color range parameters if needed. Keep *Update Min and Max?* ticked to update the range. Untick it to keep settings and continue.

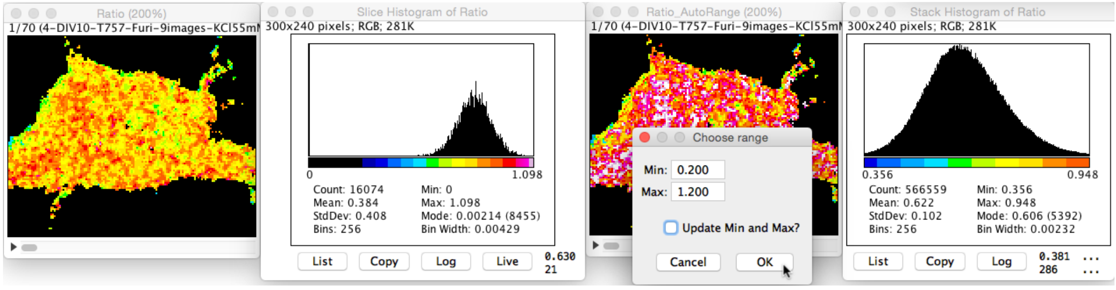

Figure 11 – Color range settings.

7. Ratiometric image is saved in the same folder as clean images, then closed.

Notes

- In the parameters option, *Selection?* greatly influence Divide behavior:

  - Image: Single: do all the steps for the image selected at step 1.

  - Image: Multi if CROP: do all the steps for the image selected at step 1, except if the image is a crop, in which case, do all the steps for all crop images detected in the folder containing the selected image.

  For the following possibilities, select a folder instead of an image at step 1.

  - Folder: CROPs only: do all the steps for all clean crops detected in the folder selected at step 1 and its subfolders.

  - Folder: All but CROPs: do all the steps for all clean images (but not crops) detected in the folder selected at step 1 and its subfolders.

  - Folder: All: do all the steps for all clean images detected in the folder selected at step 1 and its subfolders.

  Image naming convention has to be followed for images to be found in the (sub)folders.

- If *Without confirmation?* is ticked in the parameters, the above procedure will be automated, only step 1 is apparent to the user. Default settings will be used. It doesn't work with "Median" or "Mean * coefficient" thresholds which requires user interaction.

- If for some reason the ratiometric image appear in black and white, the most probable reason is that all pixels are outside of the range. To modify it, restart the tool and adjust values or use the menu Image > Adjust > Brigthness/Contrast (Shift + C), then use Set button to enter values.
- Use the menu Image > Lookup Tables > 16 colors in case the image has the wrong LUT.

4.4 Analyse Tool

1. Select Ratio image. If a RoiSet corresponding to the image is found, it is loaded in the ROI Manager.
2. If *Display weighted images?* is ticked in the parameters, the donor image is loaded and the user is asked to adjust contrast. After clicking OK, this image is replaced by the ratiometric image ponderated by the intensity range of the donor image selected by the user.

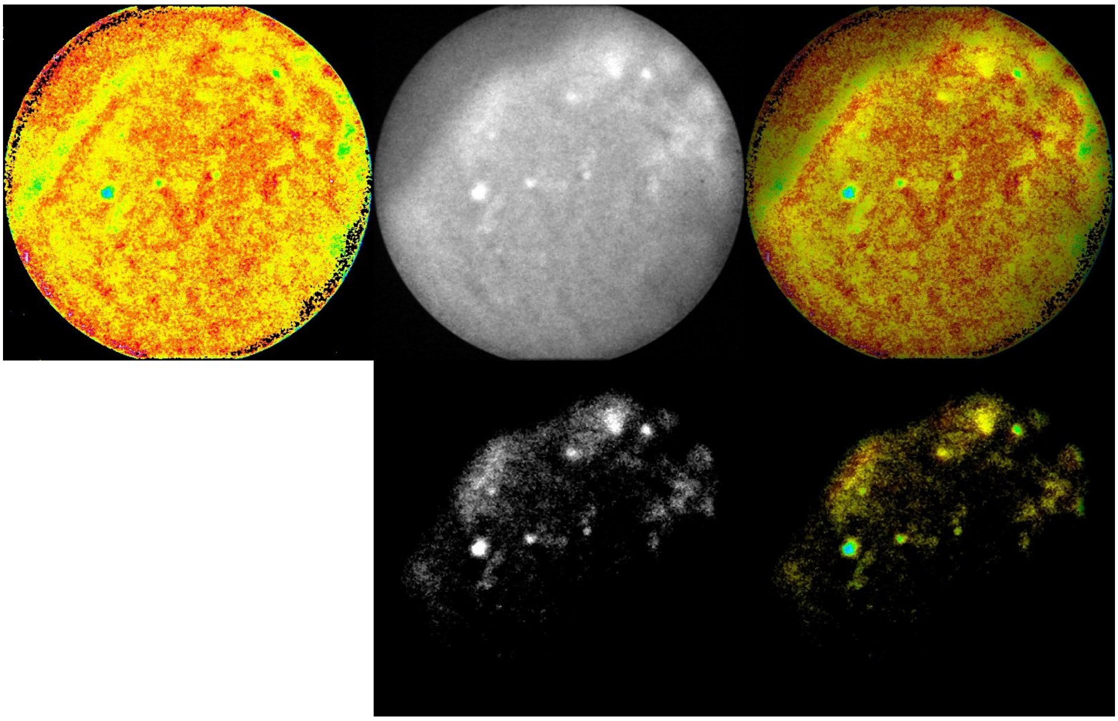

Figure 12 – Illustration of weighting process. Left: original ratiometric image. Middle: donor image with two different contrasts. Right: resulting weighting. The color of the ratiometric image is kept, but its brightness depends on the intensity of the donor image.

3. Manage regions of interest then click "OK".

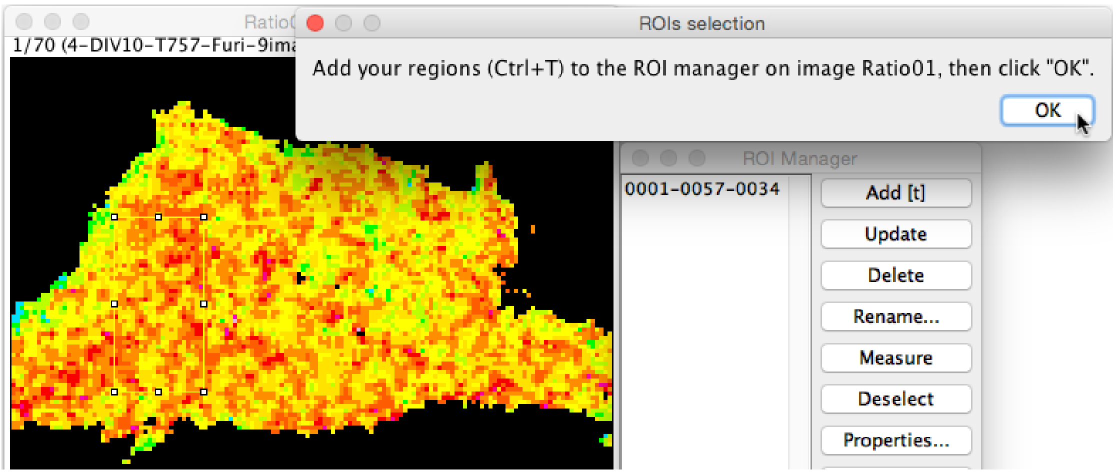

Figure 13 – Manage regions.

4. Choose to add another image or not. If yes, go back to step 1. If no, continue.

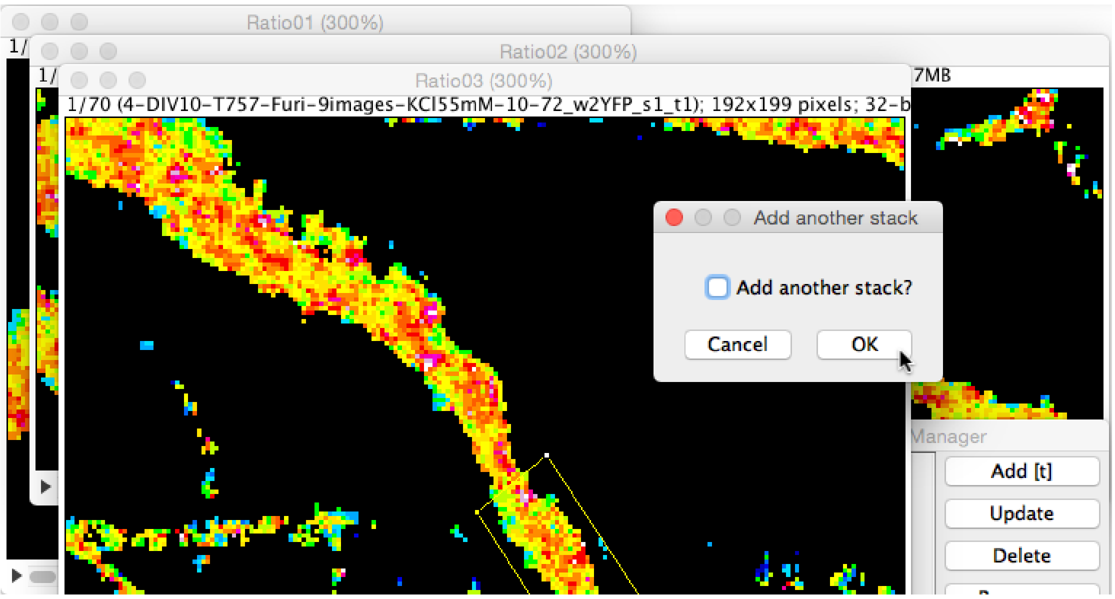

Figure 14 – Add stack?

5. Set parameters then click "OK". If *Plot Ratio vs intensity?* drop down menu is on *Vs intensity* or *Vs intensity ratio*, choose intensity image(s) and select region of interest for each Ratio image. Selected images and regions are saved and automatically loaded the next time they are used.

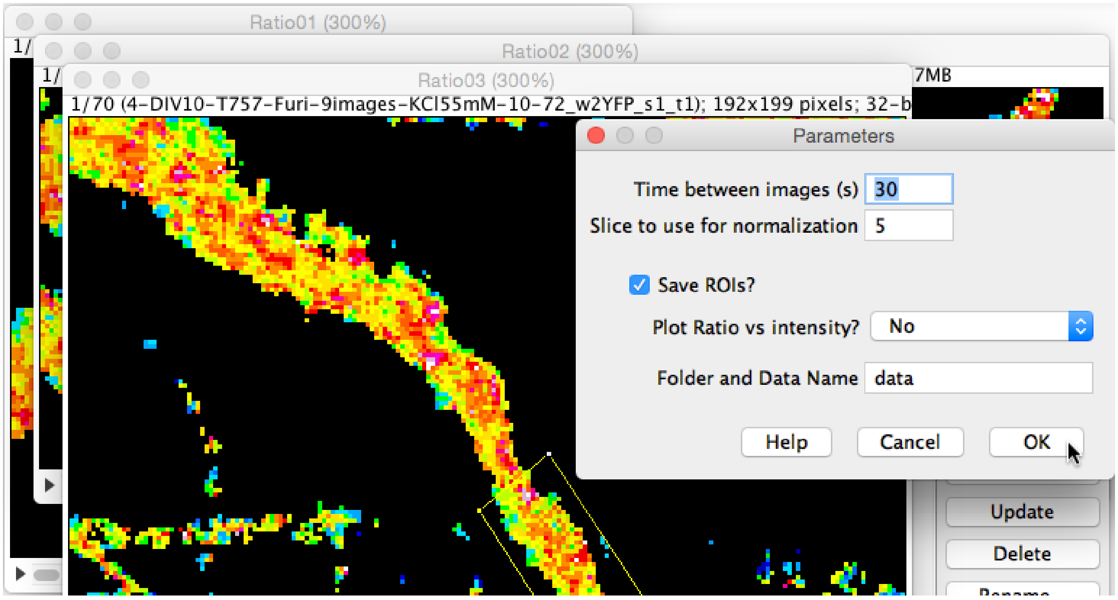

Figure 15 – Set parameters.

6. Four graphs are displayed: mean, standard deviation, normalized distribution of means and normalized distribution of standard deviation, versus time. If *Plot Ratio vs intensity?* drop down menu was on *Vs intensity* or *Vs intensity ratio*, a dynamic 3D plot window is displayed as well in case of timelapses, or a 2D plot in case of single timepoints.

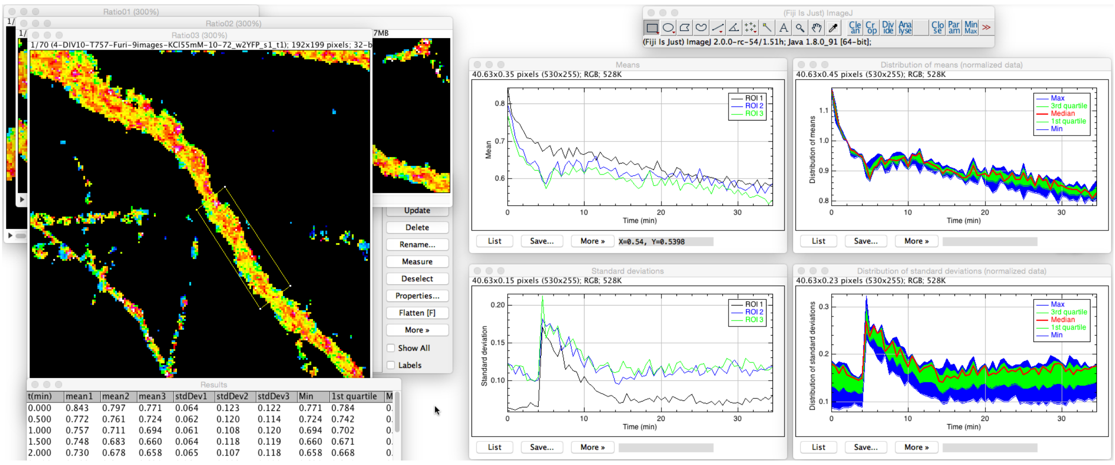

Figure 16 – Graphs.

All the data displayed in those graphs is saved in two data files, named after the character string entered in parameters' field *Folder and data name*, which will be referred to as <Data>. They are saved in a folder named <Data>, which is located in the same folder as the first ratio image used in the analysis. The file <Data>.csv (<Data>.xls) contains mean, standard deviation and distributions of regions of interest. The file <Data>Norm.csv

(<Data>Norm.xls) contains the same thing but normalized to the mean value of *Slice used to normalize*. Values from *Plot Ratio vs intensity?* are saved in <Data> file as well.

t(min)	mean1	mean2	mean3	stdDev1	stdDev2	stdDev3	Min	1st quartile	Median	3rd quartile	Max	SD Min	SD 1st quartile	SD Median	SD 3rd quartile	SD Max	Intensity_1	Intensity_2	Intensity_3
0.000	0.570	0.536	0.520	0.075	0.064	0.062	0.520	0.528	0.536	0.553	0.570	0.062	0.063	0.064	0.069	0.075	903	903	952
0.500	0.565	0.531	0.529	0.064	0.055	0.054	0.529	0.530	0.531	0.548	0.565	0.054	0.055	0.055	0.060	0.064	0	0	0
1.000	0.566	0.531	0.525	0.064	0.055	0.058	0.525	0.528	0.531	0.549	0.566	0.055	0.057	0.058	0.061	0.064	0	0	0
1.500	0.563	0.528	0.525	0.065	0.059	0.058	0.525	0.526	0.528	0.546	0.563	0.058	0.058	0.059	0.062	0.065	0	0	0
2.000	0.561	0.536	0.521	0.066	0.059	0.064	0.521	0.528	0.536	0.549	0.561	0.059	0.062	0.064	0.065	0.066	0	0	0
2.500	0.566	0.528	0.520	0.072	0.058	0.063	0.520	0.524	0.528	0.547	0.566	0.058	0.060	0.063	0.067	0.072	0	0	0
3.000	0.560	0.528	0.521	0.069	0.061	0.070	0.521	0.525	0.528	0.544	0.560	0.061	0.065	0.069	0.069	0.070	0	0	0
3.500	0.567	0.526	0.521	0.074	0.066	0.066	0.521	0.524	0.526	0.547	0.567	0.066	0.066	0.066	0.070	0.074	0	0	0
4.000	0.562	0.522	0.518	0.072	0.061	0.071	0.518	0.520	0.522	0.542	0.562	0.061	0.066	0.071	0.072	0.072	0	0	0
4.500	0.560	0.528	0.515	0.075	0.067	0.064	0.515	0.521	0.528	0.544	0.560	0.064	0.065	0.067	0.071	0.075	0	0	0
5.000	0.556	0.517	0.522	0.076	0.068	0.073	0.517	0.520	0.522	0.539	0.556	0.068	0.071	0.073	0.074	0.076	0	0	0
5.500	0.555	0.514	0.521	0.079	0.073	0.076	0.514	0.517	0.521	0.538	0.555	0.073	0.075	0.076	0.077	0.079	0	0	0
6.000	0.562	0.513	0.516	0.082	0.072	0.074	0.513	0.514	0.516	0.539	0.562	0.072	0.073	0.074	0.078	0.082	0	0	0
6.500	0.561	0.512	0.523	0.084	0.072	0.075	0.512	0.517	0.523	0.542	0.561	0.072	0.073	0.075	0.079	0.084	0	0	0
7.000	0.569	0.523	0.521	0.085	0.070	0.077	0.521	0.522	0.523	0.546	0.569	0.070	0.073	0.077	0.081	0.085	0	0	0
7.500	0.562	0.521	0.520	0.084	0.075	0.079	0.520	0.521	0.521	0.542	0.562	0.075	0.077	0.079	0.082	0.084	0	0	0
8.000	0.556	0.516	0.519	0.088	0.077	0.081	0.516	0.517	0.519	0.538	0.556	0.077	0.079	0.081	0.084	0.088	0	0	0
8.500	0.560	0.522	0.522	0.090	0.082	0.087	0.522	0.522	0.522	0.541	0.560	0.082	0.084	0.087	0.088	0.090	0	0	0
9.000	0.549	0.517	0.513	0.083	0.076	0.081	0.513	0.515	0.517	0.533	0.549	0.076	0.079	0.081	0.082	0.083	0	0	0
9.500	0.556	0.532	0.523	0.089	0.082	0.083	0.523	0.527	0.532	0.544	0.556	0.082	0.082	0.083	0.086	0.089	0	0	0

Figure 17 – Example of a data file with three regions and plot ratio vs intensity. First column is timing, followed by the three regions means, standard deviation, distribution of means, distribution of standard deviation, and finally the intensity values measured for each region.

Notes

- It is possible to replot data after modifying regions of interest (Update or Delete in the ROI Manager), and/or deleting image, then clicking on Analyse tool.
- To add a region to an existing image, open it again with "Add another stack". It will select the correct window and allow you to add more regions.
- It is possible to load a previous analysis by selecting the text file generated by the analyse tool instead of a Ratio image (see File Management).
- It is possible to pool stacks of images that don't have the same number of slices, but the distributions of value won't be computed in that case.

4.5 Param Tool

Donor and *Acceptor* fields are used to automatically detect acceptor when selecting donor, and to name images resulting from the different tools.

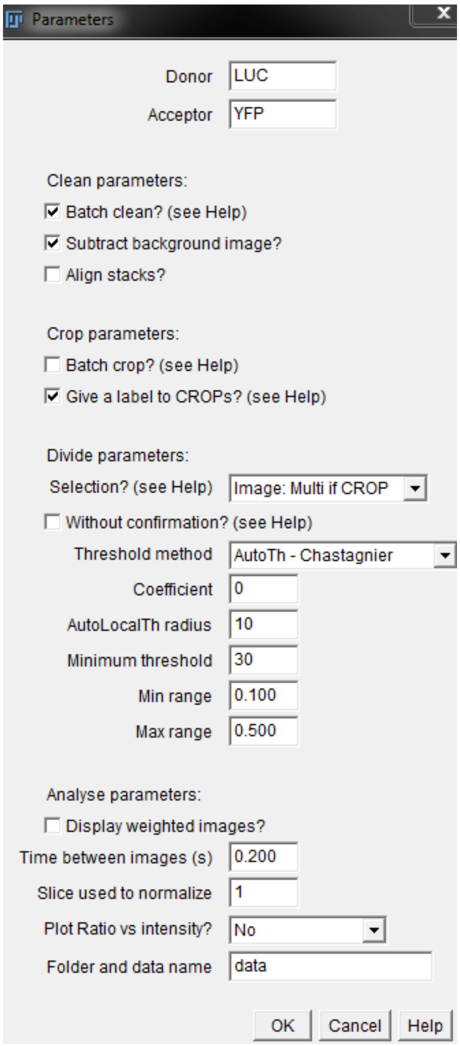

Clean parameters:

Ticking *Batch clean?* will change the behavior of Clean tool. The user will choose a folder containing raw images instead of choosing an image. All pairs of Donor/Acceptor detected inside this folder will be processed without user interaction: automatic background removal and stack alignment if ticked.

Ticking *Subtract background image?* allows the user to select reference images to remove spatially inhomogeneous background.

Align stacks? defines default value during execution.

Crop parameters:

Ticking *Batch crop?* will change the behavior of Crop tool. The user will choose a folder containing clean images instead of choosing an image. All pairs of Donor/Acceptor containing "_clean" detected inside this folder and its subfolders will be processed.

If *Give a label to CROPs?* is ticked, a text file will be generated containing the crop numbers and associated labels.

Divide parameters:

Selection? drop down menu will allow user to change Divide tool behavior:

- *Image: Single*: user selects clean image to be processed.

- *Image: Multi if CROP*: user selects clean image. If the image is a crop, all crops in the same folder will be processed. If it's not, the behavior is the same as Image: Single.

- *Folder: CROPs only*: user selects a folder. All clean crops contained in the folder and its subfolders will be processed.

- *Folder: All but CROPs*: user selects a folder. All clean images that are not crops contained in the folder and its subfolders will be processed.

- *Folder: All*: user selects a folder. All clean images contained in the folder and its subfolders will be processed. Image naming convention has to be followed for images to be found in the (sub)folders.

Ticking *Without confirmation?* will use *Threshold method*, *Coefficient*, *AutoLocalTh radius*, *Minimum threshold*, *Min range* and *Max range* values under without asking the user during execution of divide.

The threshold works as follow: for each image of donor stack, it computes the median of the area selected or mean * coefficient of the area selected or global threshold or local threshold and

put to 0 all pixels for which value is lower. After that, if *Minimum threshold* is higher than 0, set to 0 all pixels with value below.

Threshold method, *Coefficient*, *AutoLocalTh radius*, *Minimum threshold*, *Min range* and *Max range* define default values during execution.

Analyse parameters:

Ticking *Display weighted images?* will allow the user to adjust the contrast on the donor image and use that as a weighting value on the ratiometric image.

Time between images (s), *Slice used to normalize*, *Plot Ratio vs intensity?* and *Folder and data name* define default values during execution.

Setting *Plot Ratio vs intensity?* drop down menu on *Vs intensity* or *Vs intensity ratio* will allow the user to plot a 3D interactive window that displays the mean of the ROIs versus time and intensity (or intensity ratio) for time-lapse or a 2D window for single time-points.

Note : Clicking *OK* will save the parameters in Fiji's preferences file. The values will automatically be loaded the next time the macro is loaded. They are saved as well when modified in the other tools.

4.6 Min Max Tool

1. Select the folder containing the "Ratio" images.
2. Select the range to apply to the set of images.

Note: Files are overwritten with new displaying range. Values are not altered.

5 Output files structure

Following the naming convention, base images are named xxxDonor.tif and xxxAcceptor.tif. Where xxx is any character string, Donor and Acceptor are character strings from the parameters' fields Donor and Acceptor.

Clean process will create a subfolder xxx in which everything will be put, starting with clean images: xxxDonor_clean.tif and xxxAcceptor_clean.tif.

Crop process will extract a sub area of the clean images and save them as xxxCROP<i> _Donor_clean.tif and xxxCROP<i>_Acceptor_clean.tif, where <i> is crop's number. It will also save regions in cropAREAs.zip, and the crop's number with a label in file CROPs.txt if *Give a label to CROPs?* is ticked in parameters.

Divide process will create the ratiometric image xxxRatio.tif with threshold values used and/or method in text file thresholdUsed.txt. If CROP images are processed, the image and text file will be named xxxCROP<i>_Ratio.tif and thresholdUsedCROP<i>.txt, where <i> is the crop's number.

Analyse process will use the character string entered in parameters' field *Folder and data name*, which will be referred as Data in the following. A text file Data.txt is created containing the ending part of the path to images used in the analysis. It allows the user to select it instead of an image during execution of *Analyse* to quickly load all the images contained in the file and their corresponding Regions of interest. A sub-folder Data is created which will contain the following files: spreadsheets Data.csv and Data.xls containing mean, standard deviation and distributions of regions of interest, DataNorm.csv and DataNorm.xls containing the same data but normalized at a given slice, and Image<i>_ROI<j>.png for each image <i> (or Image<i>_ROI<j>-<k>.png if image contains multiple regions of interest).

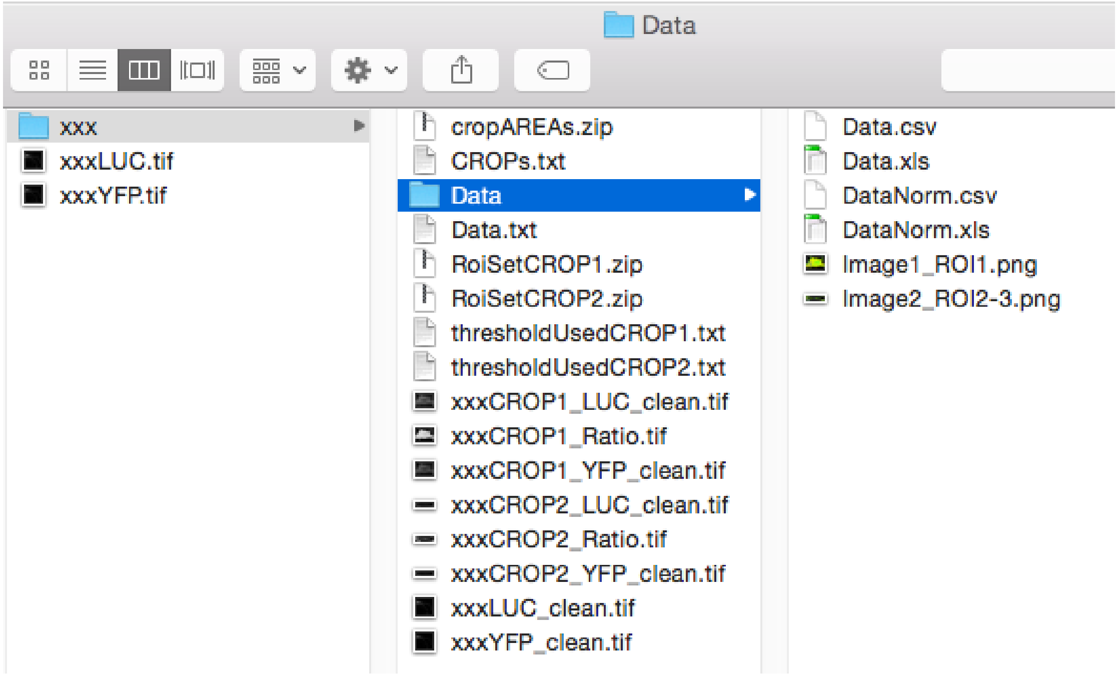

Figure 18 – Files structure.
